# Superoxide and Non-ionotropic Signaling in Neuronal Excitotoxicity

**DOI:** 10.3389/fnins.2020.00861

**Published:** 2020-09-03

**Authors:** Jiejie Wang, Raymond A. Swanson

**Affiliations:** Department of Neurology, University of California, San Francisco, and San Francisco Veterans Affairs Health Care System, San Francisco, CA, United States

**Keywords:** glutamate, glucose, Glun2B, phosphoinositol-3-kinase, metabotropic, peroxynitrite, NADPH oxidase, calcium

## Abstract

Excitotoxicity is classically attributed to Ca^2+^ influx through NMDA receptors (NMDAr), leading to production of nitric oxide by neuronal nitric oxide synthase and superoxide by mitochondria, which react to form highly cytotoxic peroxynitrite. More recent observations warrant revision of the classic view and help to explain some otherwise puzzling aspects of excitotoxic cell injury. Studies using pharmacological and genetic approaches show that superoxide produced by NMDAr activation originates primarily from NADPH oxidase rather than from mitochondria. As NADPH oxidase is localized to the plasma membrane, this also provides an explanation for the extracellular release of superoxide and cell-to-cell “spread” of excitotoxic injury observed *in vitro* and *in vivo*. The signaling pathway linking NMDAr to NADPH oxidase involves Ca^2+^ influx, phosphoinositol-3-kinase, and protein kinase Cζ, and interventions at any of these steps can prevent superoxide production and excitotoxic injury. Ca^2+^ influx specifically through NMDAr is normally required to induce excitotoxicity, through a mechanism presumed to involve privileged Ca^2+^ access to local signaling domains. However, experiments using selective blockade of the NMDAr ion channel and artificial reconstitution of Ca^2+^ by other routes indicate that the special effects of NMDAr activation are attributable instead to concurrent non-ionotropic NMDAr signaling by agonist binding to NMDAr. The non-ionotropic signaling driving NADPH oxidase activation is mediated in part by phosphoinositol-3-kinase binding to the C-terminal domain of GluN2B receptor subunits. These more recently identified aspects of excitotoxicity expand our appreciation of the complexity of excitotoxic processes and suggest novel approaches for limiting neuronal injury.

## Introduction

The term “excitotoxicity” was first used in reference to rapid neuronal death caused by glutamate receptor activation (Olney et al., 1971). The term has subsequently been used in reference to glutamate receptor-mediated cell death of other cell types, to describe more protracted cell death processes, and with activation of several different glutamate receptor subtypes. The present review focuses specifically on aspects of rapid neuronal death induced by pathological stimulation of NMDA-type glutamate receptors. This process is widely attributed to Ca^2+^ influx, leading to superoxide and nitric oxide production, which together generate the cytotoxic reactive oxygen species, peroxynitrite. However, recent studies have identified several additional complexities that challenge this classical view and identify novel ways to suppress excitotoxic neuronal death. These complexities arise from interactions between superoxide and nitric oxide, the sources of superoxide formation, and the newly appreciated role of non-ionotropic NMDA receptor (NMDAr) signaling.

## Superoxide and Nitric Oxide in Excitotoxicity: Can You Tell the Difference?

An intriguing aspect of excitotoxic cell death is that it requires the combined effects of two independently generated “gaseous” reactive oxygen species: nitric oxide and superoxide. Nitric oxide (NO) is non-polar, lipid permeable, and has a relatively long half-life and diffusion distance in brain (Pacher et al., 2007). Superoxide (O_2_^–^), by contrast, is polar, largely lipid impermeable, and has much shorter half-life and mean diffusion distance. Nitric oxide is generated in a subset of neurons by neuronal nitric oxide synthase. It is considered a reactive oxygen species (ROS) because it contains an unpaired electron, but it is not intrinsically a highly reactive molecule in biological systems. Nevertheless, studies using both pharmacological and genetic abrogation of nitric oxide production showed near-complete suppression of excitotoxic neuronal death (Dawson et al., 1991, 1996).

Superoxide, despite its name, is not a powerful oxidant and in fact behaves as a mild reductant under physiological conditions (Pacher et al., 2007). At roughly the same time that nitric oxide was identified as excitotoxic intermediate, studies using electron paramagnetic resonance and other methods demonstrated that superoxide was likewise produced by neurons during NMDAr stimulation, and that scavenging superoxide could likewise block excitotoxic death (Lafon-Cazal et al., 1993; Reynolds and Hastings, 1995; Patel et al., 1996). While at first unclear how negating either one or the other of these reactive intermediates could prevent excitotoxic injury, the observations were reconciled by the understanding that nitric oxide and superoxide combine at an extraordinarily fast rate to form the much more toxic reactive species, peroxynitrite (ONOO^–^) (Beckman et al., 1990; Radi, 2018). Peroxynitrite has subsequently become recognized as the primarily species responsible for the lipid peroxidation, DNA damage, protein nitration, and cell death that occur during excitoxicity (Lipton et al., 1994; Pacher et al., 2007; Campolo et al., 2020). Though highlighted more than 15 years ago, it remains poorly recognized how this interaction between superoxide and nitric oxide complicates the interpretation of experiments evaluating excitotoxicity, and specifically whether it is superoxide or nitric oxide that is increased (or decreased) in any particular setting (Pacher et al., 2007). Increased production of either superoxide alone or nitric oxide alone is sufficient to increase peroxynitrite production, as long as a minimal, basal level of the other species is present. Markers of peroxynitrite reaction products, such as 3-nitrotyrosine, thus increase whether the underlying cause of peroxynitrite formation is elevated superoxide, elevated nitric oxide, or both (Radi, 2018). Only with measures that assess the actual flux of these reactive intermediates can these possibilities be distinguished. As further detailed in the section “Signaling pathways underlying NOX2 activation by NMDA receptors,” the issue gains significance because interventions that are thought to act by blocking nitric oxide formation may in fact function by blocking superoxide formation, and vice versa.

## Sources of Excitotoxic Superoxide Production

Although nitric oxide is produced almost exclusively by nitric oxide synthase, superoxide can originate from multiple sources. Mitochondria can generate superoxide as a byproduct of normal respiration, by one electron addition to oxygen at the level of electron transport chain or any of several mitochondrial dehydrogenases (Andreyev et al., 2005). Importantly, mitochondria can also indirectly elevate superoxide levels when their normal superoxide scavenging functions are impaired. These scavenging functions require active regeneration of mitochondrial NADPH from NADP^+^ by electrons from mitochondrial dehydrogenases or electron transport complexes (Andreyev et al., 2005), such that impaired mitochondrial function can limit this scavenging function.

The first evidence that mitochondria could be a source of NMDA-induced superoxide elevation was based on mitochondrial localization of oxidant-sensitive fluorescent indicators together with a reduction in this mitochondrial signal with mitochondrial inhibitors (Dugan et al., 1995; Reynolds and Hastings, 1995; Bindokas et al., 1996; Duan et al., 2007). However, a reduction in the mitochondrial fluorescent dye signal in response to mitochondrial inhibitors does not establish mitochondria as the oxidant source. This is in part because the inhibitors also cause mitochondrial and plasma membrane depolarization, with resultant dye efflux and reduced signal from the mitochondria, independent of any change in actual superoxide levels (Nicholls, 2006). Mitochondrial inhibitors also reduce the oxidant scavenging capacity of mitochondria, which is extremely difficult to distinguish from a true increase in superoxide production. Moreover, the localization of oxidized dyes or any other oxidant signal to the mitochondria do not provide definitive evidence that mitochondria are the source of production, as demonstrated by oxidation of mitoSOX in neuronal mitochondria by xanthine/xanthine oxidase added to the culture medium (Johnson-Cadwell et al., 2007). Last, the calcium influx induced by NMDAr stimulation causes mitochondrial depolarization, and mitochondrial depolarization acts to reduce rather than increase superoxide production in other settings (Nicholls and Ward, 2000).

Nevertheless, many subsequent studies have provided additional, indirect evidence for mitochondria as a source of excitotoxic superoxide formation (Sengpiel et al., 1998; Starkov et al., 2004; Stanika et al., 2009; Nguyen et al., 2011; Wang et al., 2013b). Perhaps the strongest evidence comes from two studies in which genetic and pharmacological inhibition of the mitochondrial calcium uniporter was found to reduce excitotoxic death (Qiu et al., 2013; Angelova et al., 2019), however, neither of these studies evaluated actual superoxide production, and a biophysical mechanism by which Ca^2+^ movement into mitochondria drives superoxide production remains to be established.

**FIGURE 1:**
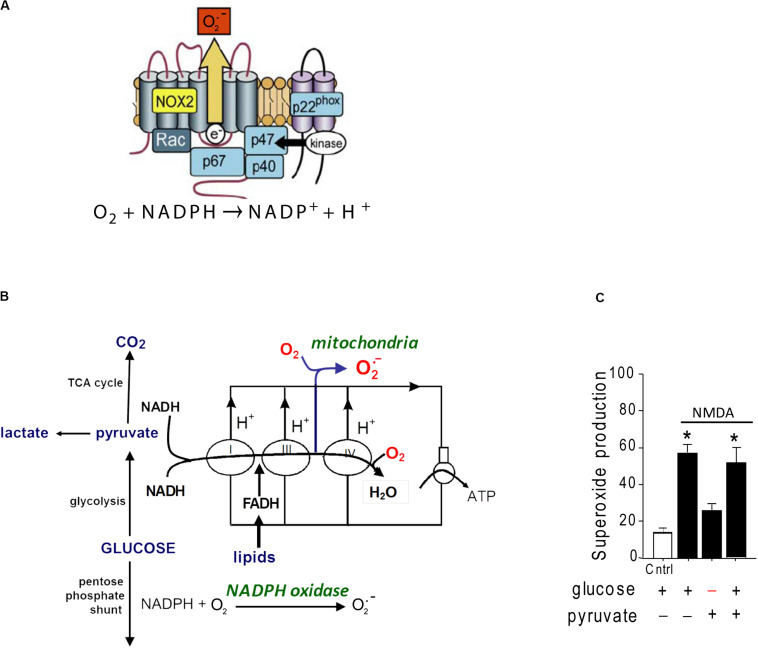
Structure and glucose dependence of NADPH oxidase. **(A)** NADPH oxidase is a multi-subunit enzyme that uses electrons derived from NADPH on one side of a membrane to generate superoxide on the other side. The NOX2 isoform of NADPH oxidase diagrammed here is the dominant form expressed by neurons. NOX2 requires activation of both the GTPase rac and the p47^*phox*^ subunit to be catalytically functional. p47^*phox*^ functions as an organizing subunit that bring the cytosolic subunits together with the membrane-bound subunits. In neurons, p47^*phox*^ is activated by phosphorylation by PKCzeta (reprinted from Bedard and Krause, 2007, with permission). **(B)** Production of superoxide by NADPH oxidase requires glucose because glucose is the requisite substrate for the pentose phosphate shunt that regenerates NADPH from NADP^+^. By contrast, superoxide production from mitochondria can be fueled by pyruvate or other non-glucose substrates. **(C)** Neurons exposed to NMDA exhibit very attenuated superoxide production when deprived of glucose, indicating NADPH oxidase rather than mitochondria as the primary source. Data are means ± SEM; *p < 0.05 vs. no glucose. Redrawn from Brennan et al. (2009).

A second source of superoxide production is the ubiquitous enzyme, nicotinamide adenine dinucleotide phosphate oxidase (abbreviated as NOX). NOX is a membrane-associated enzyme that transfers an electron from NADPH on one side of the membrane to molecular oxygen on the other side, forming O_2_^–^ (Figure 1). NOX was originally described in oocytes and neutrophils, but has subsequently been identified in most other cell types including neurons (Tejada-Simon et al., 2005; Bedard and Krause, 2007). NOX is composed of catalytic and regulatory subunits which, upon activation, coalesce with an assembly subunit at a cell membrane. The dominant NOX isoform in both neurons and neutrophils is NOX2, which contains the gp91 catalytic subunit and the p47^*phox*^ assembly subunit (Bedard and Krause, 2007). Electron micrographs show NOX2 to be localized to neuronal cell bodies and processes that also express NMDA receptors (Girouard et al., 2009; Wang et al., 2013a).

Several lines of evidence identify NOX2, rather than mitochondria, as the primary source of excitotoxic superoxide production. In cultured neurons, oxidation of the superoxide-sensitive fluorescent probe dihydroethidium is completely blocked by either pharmacological inhibition of NOX2 or genetic ablation of the p47^*phox*^ subunit (Brennan et al., 2009; Reyes et al., 2012; Brennan-Minnella et al., 2013). Pharmacologic or genetic inhibition of NOX2 likewise prevents the lipid oxidation, DNA damage, and cell death caused by NMDA receptor stimulation *in vitro* or by brain ischemia *in vivo* (Brennan et al., 2009; Girouard et al., 2009; Raz et al., 2010; Guemez-Gamboa et al., 2011; Reyes et al., 2012; Clausen et al., 2013; Koriauli et al., 2015; Ma et al., 2017). Of note, superoxide production by NOX2 has an absolute requirement for glucose, which fuels regeneration of NADPH through the pentose–phosphate pathway (Decoursey and Ligeti, 2005). By contrast, mitochondrial superoxide production can be fueled by a variety of substrates (Figure 1). Neurons deprived of glucose (and metabolically supported by pyruvate) are unable to produce superoxide in response to NMDAr activation (Brennan et al., 2009), further supporting NOX2 rather than mitochondria as the superoxide source.

The question then arises, how can it be that blocking *either* mitochondrial Ca^2+^ uptake or NOX2 activity suppresses excitotoxic superoxide production? One possibility is that excitotoxic neuronal NOX2 activation induces mitochondrial superoxide production and vice versa, such that blocking either source of superoxide also reduces superoxide production by the other. This “ROS-induced ROS” production has been demonstrated in other cell types. In vascular cells, both increased mitochondrial superoxide production (Nazarewicz et al., 2013) and depletion of mitochondrial superoxide dismutase (Dikalova et al., 2010) increase NOX2 activity. Conversely, (putative) mitochondrial superoxide production induced by angiotensin II can be blocked by suppressing NOX2 activation (Brandes, 2005; Kimura et al., 2005; Doughan et al., 2008; Dikalov et al., 2014). Evidence also suggests that superoxide produced by mitochondria can elicit further superoxide release from the densely packed mitochondria of cardiac cells (Zorov et al., 2006). To our knowledge, definitive evidence of ROS-induced ROS production has not yet been demonstrated in neurons, but for the reasons outlined earlier, this process could be central to oxidative injury in excitotoxicity.

Superoxide is also generated by other enzymes, such as xanthine oxidase, lipoxygenases, and cyclooxygenases (Snezhkina et al., 2019). However, these have not borne out as major sources of superoxide production in excitotoxicity, and in some cases the observed suppression of superoxide formation by inhibitors of these enzymes is by indirect mechanisms (as further discussed in section “Signaling Pathways Underlying NOX2 Activation by NMDA Receptors”).

## Significance of the Superoxide Production Source

**FIGURE 2:**
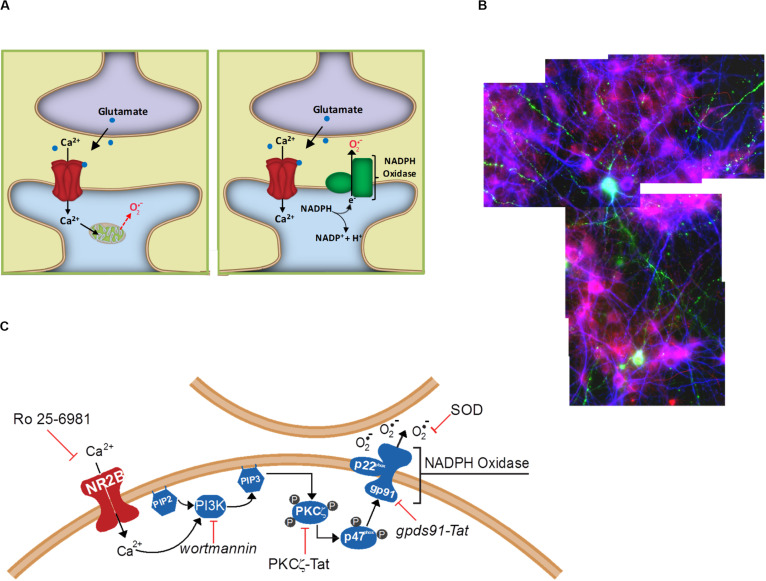
Extracellular release of superoxide by NMDA-induced NADPH oxidase activation. **(A)** Superoxide release from mitochondria will first encounter intracellular constituents of the cell in which it is enclosed, and can reach the extracellular space only be eluding cytosolic superoxide dismutase and crossing the lipid plasma membrane. By contrast, NADPH oxidase releases superoxide directly into the cytosol, where it can more readily interact with neighboring cells. **(B)** Cell-to-cell transmission of oxidative stress in neuronal cultures in which only a small subset of neurons (labeled green) contain functional NADPH oxidase. Application of NMDA to these cultures induces oxidative stress in neighboring neurons, as detected by the lipid peroxidation marker 4-hydroxynonenal (red). Cell nuclei are counterstained with DAPI (blue). Reprinted from Reyes et al. (2012). **(C)** Known steps in the signaling pathway linking NADPH oxidase to NMDAr activation. Reprinted from Minnella et al. (2018).

Researchers working with neuronal cultures have long recognized that excitotoxicity has a spreading or “bad neighborhood” effect, whereby neurons usually die in clusters and survive better when located away from other neurons. Excitotoxic death *in vivo* similarly affects contiguous populations of cells, rather than scattered individual neurons. The underlying mechanism of this spreading effect has not been established, but one possibility is the cell-to-cell effects of extracellular superoxide release. This possibility was examined in a cell culture study in which only a small sub-population of cultured neurons expressed functional NOX2 (Reyes et al., 2012). After incubation with NMDA, neurons in the vicinity of the few NOX2-competent neurons exhibited oxidative stress (lipid peroxidation), whereas neurons at a distance from these neurons did not (Figure 2). Moreover, the NOX2-competent neurons did not themselves exhibit greater oxidative stress than the NOX2-deficient cells, and the oxidative stress in all the cells was reduced by addition of superoxide dismutase to the culture medium. These observations demonstrate a trans-cellular movement of superoxide from cell to cell during NMDAr activation, and are most consistent with superoxide generated by NOX2 at the neuronal plasma membrane where it is released directly into the extracellular space. The observations are more difficult to reconcile with superoxide production by mitochondria because mitochondrial superoxide is released into the cytosol from where it can access the extracellular space only by eluding the cytosolic superoxide and H_2_O_2_ scavenging systems and crossing the lipophilic cell membrane (Figure 2). The ability of NMDAr to activate NOX2 indicates an underlying signaling pathway between them, and thus the possibility of regulating this pathway to either amplify or suppress NMDAr induced superoxide production.

## Signaling Pathways Underlying NOX2 Activation by NMDA Receptors

Several steps in this pathway have now been identified (Figure 2). NOX2 regulation is intrinsically complex, involving activation of the GTPase Rac1 and phospholipase A, permeability of a proton channel, proline isomerization, and phosphorylation of the p47^*phox*^ organizing subunit, and phosphorylation of other NOX subunits (Levy et al., 2000; Bedard and Krause, 2007; El-Benna et al., 2009; Raad et al., 2019). Of these factors, phosphorylation of p47^*phox*^ has been best characterized. p47^*phox*^-mediated assembly of the cytosolic and membrane subunits of NOX2 into a functional complex can occur only after p47^*phox*^ is phosphorylated at several serine residues, most notably ser328 (El-Benna et al., 2009). In neurons, this phosphorylation is performed primarily by protein kinase C zeta (PKCζ), an atypical, calcium-independent protein kinase. Studies using a peptide inhibitor to PKCζ showed near-complete suppression of NMDAr-induced superoxide *in vitro* and *in vivo* (Brennan et al., 2009; Brennan-Minnella et al., 2013). Interestingly, PKCζ also has a fundamental role in memory formation (Sacktor et al., 1993), which like excitotoxicity also involves superoxide signaling (Massaad and Klann, 2011).

As an atypical protein kinase C, PKCζ is not activated by Ca^2+^ or diacyl glycerol, but instead by phosphoinositol-3-phosphate, the product of phosphoinositol-3-kinase (PI3K). Accordingly, NMDAr-induced superoxide production and cell death can be blocked with the PI3K inhibitor wortmannin, and this blockade can be circumvented with either exogenous phosphoinositol-3-phosphate or with a constitutively active form of PKCζ (Brennan-Minnella et al., 2013). A more granular description of which PI3K isoform is involved in this process, and how it is activated by NMDAr activation, remains to be attained. This information would be useful, as a targeted disruption of this interaction could then dissociate superoxide production and excitotoxic injury from other aspects of NMDAr activation.

This role of PI3K in excitotoxicity appears to contrast with the other, “pro-survival” effects associated with PI3K (Brunet et al., 2001). However, neurons express several different PI3K isoforms. PI3Ks are divided into several classes on the basis of structure, regulation, and function (Hawkins et al., 2006; Gross and Bassell, 2014). Class1A PI3Ks that contain the P110γ catalytic subunit are involved in NMDAr signaling pathways (Kim et al., 2011), suggesting this may be the isoform also involved in superoxide production. On the other hand, immunoprecipitation studies have instead identified the P110β subunit in association with the NMDAr complex (Wang et al., 2012). The association between PI3K and NMDAr may be via either an adaptor protein, APPL1, which links P110β to the PSD95 complex to which synaptic NMDAr are also bound (Wang et al., 2012), or via direct binding of the PI3K p85 regulatory subunit to GluN2B (Hisatsune et al., 1999).

NMDA receptors exist as hetero-tetramers, usually with two GluN1 subunits and two GluN2 subunits. The GluN2 subunits are in turn usually of the GluN2A or GluN2B isoforms (Watanabe et al., 1993; Traynelis et al., 2010). GluN2B-containing NMDA receptors are most clearly involved in excitotoxicity (Martel et al., 2012; McQueen et al., 2017), and three lines of evidence also indicate that GluN2B is likewise specifically involved in excitotoxic superoxide production. First, superoxide production is suppressed by the putative GluN2B specific antagonist Ro 25-6981 (Brennan-Minnella et al., 2013) and by depletion of GluN2B from the NMDAr complexes (Minnella et al., 2018). Second, replacement of the GluN2A intracellular C-terminal with the GluN2B intracellular C-terminal leads to superoxide formation from cells expressing these chimeric GluN2A/B subunits (Minnella et al., 2018). Third, a synthetic peptide that blocks GluN2B binding to the multiprotein PSD-95 complex also blocks excitotoxic superoxide production (Chen et al., 2015).

Of note, this peptide (termed “Tat-NR2B9c” or “NA-1”) was originally designed to block NMDAr-induced nitric oxide production by displacing GluN2B from the PSD95, with which neuronal nitric oxide synthase is associated (Sattler et al., 1999; Aarts et al., 2002). Measures of cGMP formation in the presence of Tat-NR2B9c confirm that it effectively reduces NMDAr-induced NO production, and the salutary effects on this peptide are widely attributed to this effect. However, given that the peptide also blocks NMDAr-induced NOX2 activation (also by displacing GluN2B from PSD95; Chen et al., 2015), and given that reductions in either nitric oxide or superoxide reduce peroxynitrite formation, it is equally possible that the neuroprotective effect of Tat-NR2B9c is attributable to instead to reduced superoxide production (or to the combined effects of reducing both superoxide and nitric oxide production). The Tat-NR2B9c peptide is now being used in clinical trials for ischemic stroke (Hill et al., 2020).

## Route of Calcium Influx in Excitotoxic Superoxide Production

While several aspects of the signaling pathway linking NMDAr to NOX2 activation remain uncertain, the fundamental question of which step is Ca^2+^ dependent remains unresolved. Other Ca^2+^-dependent NMDAr signaling events have been shown to be mediated by calcium/calmodulin-dependent protein kinases (Bayer and Schulman, 2019), and in particular CaMKII, which binds to NMDAr. Inhibition of CaMKII has been shown to block excitotoxicity, although with a complex temporal pattern (Ashpole and Hudmon, 2011). Several GluN2B phosphorylation sites regulate NMDA receptor trafficking, including tyrosines 1070, 1472, and 1480 (Prybylowski et al., 2005; Sanz-Clemente et al., 2010; Lu et al., 2015; Chiu et al., 2019). Of these, deletion of the 1472 tyrosine residue has been shown to suppress NMDAr-induced superoxide production (Knox et al., 2014). It is also possible that Ca^2+^ influx is instead in involved in one of the other processes required for NOX2 activation, such as Rac1 activation (Puri, 2020), phospholipase activation, or p47^*phox*^ proline isomerization.

A related issue is the longstanding controversy as to whether there is a “special” role for Ca^2+^ entering via NMDAr or whether only the magnitude of Ca^2+^ increase is important. Published data support both sides of this issue (e.g., Tymianski et al., 1993; Sattler et al., 1998; Stanika et al., 2009, 2012). This controversy may be resolved by a study from our laboratory that compared effects of Ca^2+^ influx induced by the calcium ionophore ionomycin with that of Ca^2+^ influx induced by NMDAr activation. Whereas NMDAr activation induced NOX2 activation, ionomycin did not, under conditions in which ionomycin was titrated to match the intracellular Ca^2+^ elevations induced by NMDAr activation (Minnella et al., 2018). Very similar effects were observed using activation of voltage-gated calcium channels to induce Ca^2+^ influx. However, ionomycin used at concentrations that raised intracellular Ca^2+^ well above the levels induced by NMDA did induce superoxide production, and this mode of superoxide production was not blocked by NOX2 inhibition. These observations suggest that Ca^2+^ influx by routes other than NMDAr can induce superoxide production only if the resulting Ca^2+^ elevations are far higher than achieved by NMDAr activation. They also provide support for a special effect of Ca^2+^ influx via NMDAr, as this induced superoxide production, oxidative stress, and cell death at intracellular Ca^2+^ levels that did none of these things when produced by ionomycin or voltage-gated calcium channels. However, as outlined later, these observations may alternatively be explained by concurrent, non-ionotropic effects of NMDAr stimulation that are not engaged during Ca^2+^ influx by other routes.

## Non-Ionotropic NMDAr Signaling in Excitotoxicity

Ca^2+^ influx through NMDAr ion channels has long been established as a necessary event in excitotoxicity (Choi, 1985), as evidenced by the potent cytoprotective effects of drugs such as MK801 that block NMDAr ion channels. As noted earlier, Ca^2+^ elevations achieved by influx routes other than NMDAr (of comparable magnitude) do not produce nitric oxide production, superoxide production, or excitotoxic cell death (Tymianski et al., 1993; Sattler et al., 1998; Minnella et al., 2018). These observations are widely interpreted as evidence for a privileged access to certain local signaling pathways by Ca^2+^ influx specifically through NMDAr, however, it has recently been recognized that these observations can alternatively be explained by engagement of non-ionotropic NMDAr signaling in parallel to NMDAr-induced Ca^2+^ influx.

**FIGURE 3:**
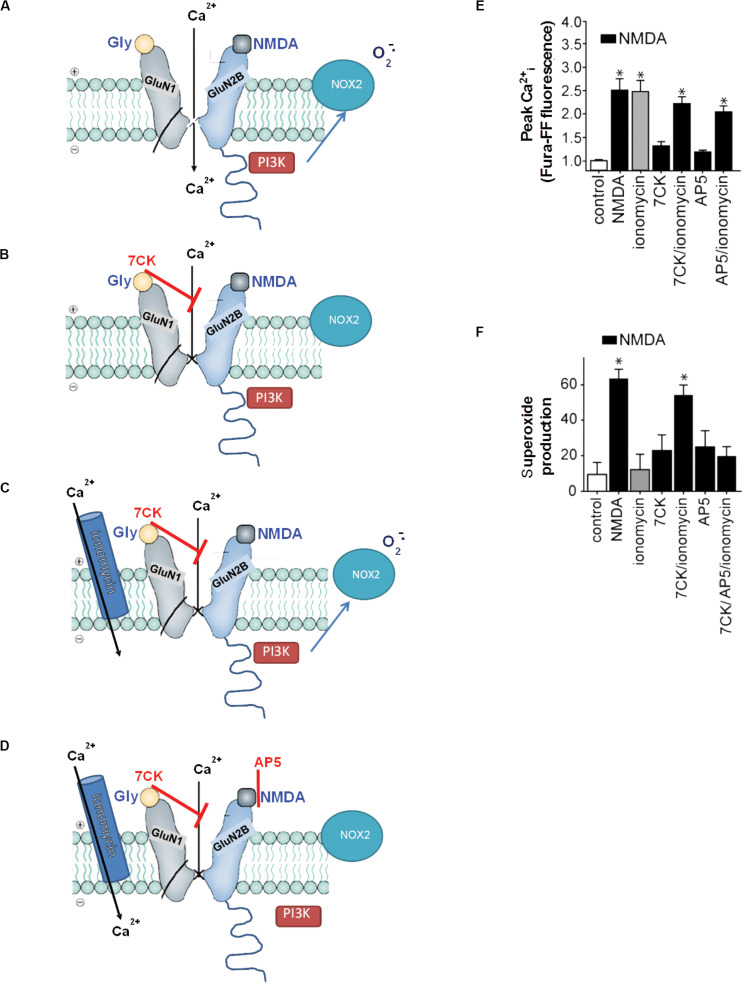
Non-ionotropic signaling in excitotoxicity. **(A)** Binding of the agonists glycine and NMDA to an NMDAr containing a GluN2B subunit triggers Ca^2+^ influx through the NMDAr channel and subsequent superoxide production by NOX2. **(B)** If the Ca^2+^ influx is blocked by the NMDAr glycine site antagonist 7-chlorokynurenic acid (7CK), there is no superoxide formation despite NMDA binding. **(C)** If, in addition, ionomycin is used to reconstitute the Ca^2+^ influx, superoxide is again produced by NOX2. **(D)** Ionomycin does not induce superoxide production when NMDA binding to GluN2B is blocked by (2R)-amino-5-phosphonopentanoate (AP5). Note the association of PI3K with the GluN2 subunit of the NMDAr requires ligand binding to the GluN2 subunit, but does not require Ca^2+^ influx. **(E,F)** Quantified measure of intracellular Ca^2+^ elevations and superoxide production under the conditions diagrammed in **(A–D)**. Data are means ± SEM; *p < 0.05 vs. control. Reprinted from Minnella et al. (2018), with modifications.

“Non-ionotropic” receptor signaling refers to signal transduction events triggered by transmembrane conformational changes rather than by ion flux. Non-ionotropic signaling by NMDAr was first identified by Westbrook and colleagues, who found that agonist binding independent of ion flux could downregulate GluN1/GluN2A receptor function through a process involving dephosphorylation of GluN1 tyrosine residues (Vissel et al., 2001). Subsequent studies showed that non-ionotropic NMDAr signaling also mediated long-term depression, p38 phosphorylation (Nabavi et al., 2013; Tamburri et al., 2013), and dendritic spine shrinkage (Stein et al., 2020). These studies identified non-ionotropic signaling by selectively blocking flux through the NMDAr ion channel while preserving agonist binding to the NMDAr itself (Figure 3). Using this same approach, Minnella and colleagues showed that NMDAr channel blockers prevented the production of superoxide (as expected), and that superoxide production and excitotoxicity were restored when Ca^2+^ influx was reconstituted through ionomycin or voltage-gated calcium channels during NMDAr stimulation (Minnella et al., 2018). Crucially, the Ca^2+^ influx induced by these routes failed to induce superoxide production in the absence of concurrent agonist binding to NMDAr, thus demonstrating a requisite role for non-ionotropic NMDAr binding in addition to Ca^2+^ influx (Figure 3). Ligand binding to NMDAr in the absence of Ca^2+^ influx increased PI3K association with GluN2B (as assessed by immunoprecipitation), thus suggesting a mechanism coupling non-ionotropic signaling to NOX2 activation.

A second and apparently independent role of metabotropic signaling in excitotoxicity involves pannexin channel opening. When activated, pannexin ion channels become permeable to Na^+^, Ca^2+^, and other ions and molecules (Yeung et al., 2020). Thompson and colleagues have shown that sustained NMDAr activation can induce pannexin-1 channel activation in the absence of ion flux through the NMDAr ion channel, with the resulting Ca^2+^ and Na^+^ influx essentially amplify the elevations caused by ion flux through the NMDAr itself. A synthetic peptide that prevents pannexin-1 channel activation by preventing its phosphorylation by Src kinase attenuates NMDAr-induced Ca^2+^ elevations and neuronal death (Weilinger et al., 2012, 2016). The relationship between this non-ionotropic signaling pathway and non-ionotropic NOX2 activation has not yet been resolved. However, NOX2 activation does not appear to be downstream of pannexin opening because the pannexin-1 inhibitor probenecid does not suppress NMDAr-induced superoxide production (J. Wang and R. Swanson, unpublished results).

## Summary

The early observations that identified key roles for Ca^2+^, superoxide, and nitric oxide in excitotoxicity have stood the test of time, but subsequent observations have identified complexities that both expand our understanding of this process and open additional questions. Among these complexities is that elevations in either superoxide or nitric oxide levels can drive production of peroxynitrite, such that it is rarely possible to ascertain which one of these ROS is driving excitotoxic injury. It has also been demonstrated that superoxide production induced by NMDAr stimulation is generated primarily by NOX2, rather than mitochondria, but it remains uncertain whether this superoxide signal may be amplified by resultant mitochondrial dysfunction. The identification of NOX2 as the primary source of superoxide and provides an explanation for the extracellular actions of superoxide in excitoxicity, and also indicates the existence of a signaling cascade linking NMDAr to NADPH oxidase. This signaling cascade has been shown to include Ca^2+^ influx, PI3K activation, and PKCζ activation, but the nature of the Ca^2+^-dependent step remains to be established. Notably, Ca^2+^ influx alone is not sufficient to induce superoxide formation or excitotoxicity, and recent studies suggest that these processes also require concomitant non-ionotropic signaling induced by agonist binding to NMDAr.

## Author Contributions

RS and JW both contributed to the literature research and writing for this review. Both authors contributed to the article and approved the submitted version.

## Conflict of Interest

The authors declare that the research was conducted in the absence of any commercial or financial relationships that could be construed as a potential conflict of interest.

## References

[B1] Aarts M. Liu Y. Liu L. Besshoh S. Arundine M. Gurd J. W. (2002). Treatment of ischemic brain damage by perturbing NMDA receptor- PSD-95 protein interactions. *Science* 298 846–850. 10.1126/science.1072873 12399596

[B2] Andreyev A. Y. Kushnareva Y. E. Starkov A. A. (2005). Mitochondrial metabolism of reactive oxygen species. *Biochemistry (Mosc)* 70 200–214. 15807660 10.1007/s10541-005-0102-7

[B3] Angelova P. R. Vinogradova D. Neganova M. E. Serkova T. P. Sokolov V. V. Bachurin S. O. (2019). Pharmacological sequestration of mitochondrial calcium uptake protects neurons against glutamate excitotoxicity. *Mol. Neurobiol.* 56 2244–2255. 10.1007/s12035-018-1204-8 30008072 PMC6394642

[B4] Ashpole N. M. Hudmon A. (2011). Excitotoxic neuroprotection and vulnerability with CaMKII inhibition. *Mol. Cell Neurosci.* 46 720–730. 10.1016/j.mcn.2011.02.003 21316454

[B5] Bayer K. U. Schulman H. (2019). CaM kinase: still inspiring at 40. *Neuron* 103 380–394. 10.1016/j.neuron.2019.05.033 31394063 PMC6688632

[B6] Beckman J. S. Beckman T. W. Chen J. Marshall P. A. Freeman B. A. (1990). Apparent hydroxyl radical production by peroxynitrite: implications for endothelial injury from nitric oxide and superoxide. *Proc. Natl. Acad. Sci. U.S.A.* 87 1620–1624. 10.1073/pnas.87.4.1620 2154753 PMC53527

[B7] Bedard K. Krause K. H. (2007). The NOX family of ROS-generating NADPH oxidases: physiology and pathophysiology. *Physiol. Rev.* 87 245–313. 10.1152/physrev.00044.2005 17237347

[B8] Bindokas V. P. Jordan J. Lee C. C. Miller R. J. (1996). Superoxide production in rat hippocampal neurons: selective imaging with hydroethidine. *J. Neurosci.* 16 1324–1336. 10.1523/jneurosci.16-04-01324.1996 8778284 PMC6578569

[B9] Brandes R. P. (2005). Triggering mitochondrial radical release: a new function for NADPH oxidases. *Hypertension* 45 847–848. 10.1161/01.hyp.0000165019.32059.b2 15837827

[B10] Brennan A. M. Suh S. W. Won S. J. Narasimhan P. Kauppinen T. M. Lee H. (2009). NADPH oxidase is the primary source of superoxide induced by NMDA receptor activation. *Nat. Neurosci.* 12 857–863. 10.1038/nn.2334 19503084 PMC2746760

[B11] Brennan-Minnella A. M. Shen Y. El-Benna J. Swanson R. A. (2013). Phosphoinositide 3-kinase couples NMDA receptors to superoxide release in excitotoxic neuronal death. *Cell Death Dis.* 4:e580. 10.1038/cddis.2013.111 23559014 PMC3641334

[B12] Brunet A. Datta S. R. Greenberg M. E. (2001). Transcription-dependent and -independent control of neuronal survival by the PI3K-Akt signaling pathway. *Curr. Opin. Neurobiol.* 11 297–305. 10.1016/s0959-4388(00)00211-7 11399427

[B13] Campolo N. Issoglio F. M. Estrin D. A. Bartesaghi S. Radi R. (2020). 3-Nitrotyrosine and related derivatives in proteins: precursors, radical intermediates and impact in function. *Essays Biochem.* 64 111–133. 10.1042/ebc20190052 32016371

[B14] Chen Y. Brennan-Minnella A. M. Sheth S. El-Benna J. Swanson R. A. (2015). Tat-NR2B9c prevents excitotoxic neuronal superoxide production. *J. Cereb. Blood Flow Metab.* 35 739–742. 10.1038/jcbfm.2015.16 25669908 PMC4420863

[B15] Chiu A. M. Wang J. Fiske M. P. Hubalkova P. Barse L. Gray J. A. (2019). NMDAR-Activated PP1 dephosphorylates GluN2B to modulate NMDAR synaptic content. *Cell Rep.* 28 332–341.e5.. 31291571 10.1016/j.celrep.2019.06.030 PMC6639021

[B16] Choi D. W. (1985). Glutamate neurotoxicity in cortical cell culture is calcium dependent. *Neurosci. Lett.* 58 293–297. 10.1016/0304-3940(85)90069-2 2413399

[B17] Clausen A. Mcclanahan T. Ji S. G. Weiss J. H. (2013). Mechanisms of rapid reactive oxygen species generation in response to cytosolic Ca2+ or Zn2+ loads in cortical neurons. *PLoS One* 8:e83347. 10.1371/journal.pone.0083347 24340096 PMC3858366

[B18] Dawson V. L. Dawson T. M. London E. D. Bredt D. S. Snyder S. H. (1991). Nitric oxide mediates glutamate neurotoxicity in primary cortical cultures. *Proc. Natl. Acad. Sci. U.S.A.* 88 6368–6371. 10.1073/pnas.88.14.6368 1648740 PMC52084

[B19] Dawson V. L. Kizushi V. M. Huang P. L. Snyder S. H. Dawson T. M. (1996). Resistance to neurotoxicity in cortical cultures from neuronal nitric oxide synthase-deficient mice. *J. Neurosci.* 16 2479–2487. 10.1523/jneurosci.16-08-02479.1996 8786424 PMC6578778

[B20] Decoursey T. E. Ligeti E. (2005). Regulation and termination of NADPH oxidase activity. *Cell Mol. Life Sci.* 62 2173–2193. 10.1007/s00018-005-5177-1 16132232 PMC11139152

[B21] Dikalov S. I. Nazarewicz R. R. Bikineyeva A. Hilenski L. Lassegue B. Griendling K. K. (2014). Nox2-induced production of mitochondrial superoxide in angiotensin II-mediated endothelial oxidative stress and hypertension. *Antioxid. Redox. Signal* 20 281–294. 10.1089/ars.2012.4918 24053613 PMC3887459

[B22] Dikalova A. E. Bikineyeva A. T. Budzyn K. Nazarewicz R. R. Mccann L. Lewis W. (2010). Therapeutic targeting of mitochondrial superoxide in hypertension. *Circ. Res.* 107 106–116. 10.1161/circresaha.109.214601 20448215 PMC2901409

[B23] Doughan A. K. Harrison D. G. Dikalov S. I. (2008). Molecular mechanisms of angiotensin II-mediated mitochondrial dysfunction: linking mitochondrial oxidative damage and vascular endothelial dysfunction. *Circ. Res.* 102 488–496. 10.1161/circresaha.107.162800 18096818

[B24] Duan Y. Gross R. A. Sheu S. S. (2007). Ca2+-dependent generation of mitochondrial reactive oxygen species serves as a signal for poly(ADP-ribose) polymerase-1 activation during glutamate excitotoxicity. *J. Physiol.* 585 741–758. 10.1113/jphysiol.2007.145409 17947304 PMC2375529

[B25] Dugan L. L. Sensi S. L. Canzoniero L. M. Handran S. D. Rothman S. M. Lin T. S. (1995). Mitochondrial production of reactive oxygen species in cortical neurons following exposure to N-methyl-D-aspartate. *J. Neurosci.* 15 6377–6388. 10.1523/jneurosci.15-10-06377.1995 7472402 PMC6578019

[B26] El-Benna J. Dang P. M. Gougerot-Pocidalo M. A. Marie J. C. Braut-Boucher F. (2009). p47phox, the phagocyte NADPH oxidase/NOX2 organizer: structure, phosphorylation and implication in diseases. *Exp. Mol. Med.* 41 217–225. 19372727 10.3858/emm.2009.41.4.058 PMC2679237

[B27] Girouard H. Wang G. Gallo E. F. Anrather J. Zhou P. Pickel V. M. (2009). NMDA receptor activation increases free radical production through nitric oxide and NOX2. *J. Neurosci.* 29 2545–2552. 10.1523/jneurosci.0133-09.2009 19244529 PMC2669930

[B28] Gross C. Bassell G. J. (2014). Neuron-specific regulation of class I PI3K catalytic subunits and their dysfunction in brain disorders. *Front. Mol. Neurosci.* 7:12. 10.3389/fnmol.2014.00012 24592210 PMC3923137

[B29] Guemez-Gamboa A. Estrada-Sanchez A. M. Montiel T. Paramo B. Massieu L. Moran J. (2011). Activation of NOX2 by the stimulation of ionotropic and metabotropic glutamate receptors contributes to glutamate neurotoxicity in vivo through the production of reactive oxygen species and calpain activation. *J. Neuropathol. Exp. Neurol.* 70 1020–1035. 10.1097/nen.0b013e3182358e4e 22002428

[B30] Hawkins P. T. Anderson K. E. Davidson K. Stephens L. R. (2006). Signalling through Class I PI3Ks in mammalian cells. *Biochem. Soc. Trans.* 34 647–662. 10.1042/bst0340647 17052169

[B31] Hill M. D. Goyal M. Menon B. K. Nogueira R. G. Mctaggart R. A. Demchuk A. M. (2020). Efficacy and safety of nerinetide for the treatment of acute ischaemic stroke (ESCAPE-NA1): a multicentre, double-blind, randomised controlled trial. *Lancet* 395 878–887. 32087818 10.1016/S0140-6736(20)30258-0

[B32] Hisatsune C. Umemori H. Mishina M. Yamamoto T. (1999). Phosphorylation-dependent interaction of the N-methyl-D-aspartate receptor epsilon 2 subunit with phosphatidylinositol 3-kinase. *Genes Cells* 4 657–666. 10.1046/j.1365-2443.1999.00287.x 10620012

[B33] Johnson-Cadwell L. I. Jekabsons M. B. Wang A. Polster B. M. Nicholls D. G. (2007). ‘Mild Uncoupling’ does not decrease mitochondrial superoxide levels in cultured cerebellar granule neurons but decreases spare respiratory capacity and increases toxicity to glutamate and oxidative stress. *J. Neurochem.* 101 1619–1631. 10.1111/j.1471-4159.2007.04516.x 17437552

[B34] Kim J. I. Lee H. R. Sim S. E. Baek J. Yu N. K. Choi J. H. (2011). PI3Kgamma is required for NMDA receptor-dependent long-term depression and behavioral flexibility. *Nat. Neurosci.* 14 1447–1454. 22019731 10.1038/nn.2937

[B35] Kimura S. Zhang G. X. Nishiyama A. Shokoji T. Yao L. Fan Y. Y. (2005). Role of NAD(P)H oxidase- and mitochondria-derived reactive oxygen species in cardioprotection of ischemic reperfusion injury by angiotensin II. *Hypertension* 45 860–866. 10.1161/01.hyp.0000163462.98381.7f 15824196

[B36] Knox R. Brennan-Minnella A. M. Lu F. Yang D. Nakazawa T. Yamamoto T. (2014). NR2B phosphorylation at tyrosine 1472 contributes to brain injury in a rodent model of neonatal hypoxia-ischemia. *Stroke* 45 3040–3047. 10.1161/strokeaha.114.006170 25158771 PMC4175024

[B37] Koriauli S. Natsvlishvili N. Barbakadze T. Mikeladze D. (2015). Knockdown of interleukin-10 induces the redistribution of sigma1-receptor and increases the glutamate-dependent NADPH-oxidase activity in mouse brain neurons. *Biol. Res.* 48 55. 10.1186/s40659-015-0048-1 PMC4599652 26453192

[B38] Lafon-Cazal M. Pietri S. Culcasi M. Bockaert J. (1993). NMDA-dependent superoxide production and neurotoxicity. *Nature* 364 535–537. 10.1038/364535a0 7687749

[B39] Levy R. Lowenthal A. Dana R. (2000). Cytosolic phospholipase A2 is required for the activation of the NADPH oxidase associated H+ channel in phagocyte-like cells. *Adv. Exp. Med. Biol.* 479 125–135. 10.1007/0-306-46831-x_11 10897415

[B40] Lipton S. A. Singel D. J. Stamler J. S. (1994). Nitric oxide in the central nervous system. *Prog. Brain Res.* 103 359–364. 7886218 10.1016/s0079-6123(08)61149-8

[B41] Lu W. Fang W. Li J. Zhang B. Yang Q. Yan X. (2015). Phosphorylation of Tyrosine 1070 at the GluN2B subunit is regulated by synaptic activity and critical for surface expression of N-Methyl-D-aspartate (n.d.) receptors. *J. Biol. Chem.* 290 22945–22954. 10.1074/jbc.m115.663450 26229100 PMC4645598

[B42] Ma M. W. Wang J. Zhang Q. Wang R. Dhandapani K. M. Vadlamudi R. K. (2017). NADPH oxidase in brain injury and neurodegenerative disorders. *Mol. Neurodegener.* 12 7. 10.1186/s13024-017-0150-7 PMC5240251 28095923

[B43] Martel M. A. Ryan T. J. Bell K. F. Fowler J. H. Mcmahon A. Al-Mubarak B. (2012). The subtype of GluN2 C-terminal domain determines the response to excitotoxic insults. *Neuron* 74 543–556. 10.1016/j.neuron.2012.03.021 22578505 PMC3398391

[B44] Massaad C. A. Klann E. (2011). Reactive oxygen species in the regulation of synaptic plasticity and memory. *Antioxid. Redox. Signal* 14 2013–2054. 10.1089/ars.2010.3208 20649473 PMC3078504

[B45] McQueen J. Ryan T. J. Mckay S. Marwick K. Baxter P. Carpanini S. M. (2017). Pro-death NMDA receptor signaling is promoted by the GluN2B C-terminus independently of Dapk1. *Elife* 6:e17161. 10.7554/eLife.17161 PMC5544426 28731405

[B46] Minnella A. M. Zhao J. X. Jiang X. Jakobsen E. Lu F. Wu L. (2018). Excitotoxic superoxide production and neuronal death require both ionotropic and non-ionotropic NMDA receptor signaling. *Sci. Rep.* 8:17522. 10.1038/s41598-018-35725-5 PMC6269523 30504838

[B47] Nabavi S. Kessels H. W. Alfonso S. Aow J. Fox R. Malinow R. (2013). Metabotropic NMDA receptor function is required for NMDA receptor-dependent long-term depression. *Proc. Natl. Acad. Sci. U.S.A.* 110 4027–4032. 10.1073/pnas.1219454110 23431133 PMC3593861

[B48] Nazarewicz R. R. Dikalova A. E. Bikineyeva A. Dikalov S. I. (2013). Nox2 as a potential target of mitochondrial superoxide and its role in endothelial oxidative stress. *Am. J. Physiol. Heart Circ. Physiol.* 305 H1131–H1140. 23955717 10.1152/ajpheart.00063.2013 PMC3798790

[B49] Nguyen D. Alavi M. V. Kim K. Y. Kang T. Scott R. T. Noh Y. H. (2011). A new vicious cycle involving glutamate excitotoxicity, oxidative stress and mitochondrial dynamics. *Cell Death Dis.* 2:e240. 10.1038/cddis.2011.117 22158479 PMC3252734

[B50] Nicholls D. G. (2006). Simultaneous monitoring of ionophore- and inhibitor-mediated plasma and mitochondrial membrane potential changes in cultured neurons. *J. Biol. Chem.* 281 14864–14874. 10.1074/jbc.m510916200 16551630

[B51] Nicholls D. G. Ward M. W. (2000). Mitochondrial membrane potential and neuronal glutamate excitotoxicity: mortality and millivolts. *Trends Neurosci.* 23 166–174. 10.1016/s0166-2236(99)01534-9 10717676

[B52] Olney J. W. Ho O. L. Rhee V. (1971). Cytotoxic effects of acidic and sulphur containing amino acids on the infant mouse central nervous system. *Exp. Brain Res.* 14 61–76. 5157537 10.1007/BF00234911

[B53] Pacher P. Beckman J. S. Liaudet L. (2007). Nitric oxide and peroxynitrite in health and disease. *Physiol. Rev.* 87 315–424. 17237348 10.1152/physrev.00029.2006 PMC2248324

[B54] Patel M. Day B. J. Crapo J. D. Fridovich I. Mcnamara J. O. (1996). Requirement for superoxide in excitotoxic cell death. *Neuron* 16 345–355. 10.1016/s0896-6273(00)80052-5 8789949

[B55] Prybylowski K. Chang K. Sans N. Kan L. Vicini S. Wenthold R. J. (2005). The synaptic localization of NR2B-containing NMDA receptors is controlled by interactions with PDZ proteins and AP-2. *Neuron* 47 845–857. 10.1016/j.neuron.2005.08.016 16157279 PMC1350965

[B56] Puri B. K. (2020). Calcium signaling and gene expression. *Adv. Exp. Med. Biol.* 1131 537–545. 10.1007/978-3-030-12457-1_22 31646525

[B57] Qiu J. Tan Y. W. Hagenston A. M. Martel M. A. Kneisel N. Skehel P. A. (2013). Mitochondrial calcium uniporter Mcu controls excitotoxicity and is transcriptionally repressed by neuroprotective nuclear calcium signals. *Nat. Commun.* 4:2034. 10.1038/ncomms3034 PMC3709514 23774321

[B58] Raad H. Derkawi R. A. Tlili A. Belambri S. A. Dang P. M. El-Benna J. (2019). Phosphorylation of gp91(phox)/NOX2 in Human Neutrophils. *Methods Mol. Biol.* 1982 341–352. 31172483 10.1007/978-1-4939-9424-3_21

[B59] Radi R. (2018). Oxygen radicals, nitric oxide, and peroxynitrite: redox pathways in molecular medicine. *Proc. Natl. Acad. Sci. U.S.A.* 115 5839–5848. 10.1073/pnas.1804932115 29802228 PMC6003358

[B60] Raz L. Zhang Q. G. Zhou C. F. Han D. Gulati P. Yang L. C. (2010). Role of Rac1 GTPase in NADPH oxidase activation and cognitive impairment following cerebral ischemia in the rat. *PLoS One* 5:e12606. 10.1371/journal.pone.0012606 20830300 PMC2935374

[B61] Reyes R. C. Brennan A. M. Shen Y. Baldwin Y. Swanson R. A. (2012). Activation of neuronal NMDA receptors induces superoxide-mediated oxidative stress in neighboring neurons and astrocytes. *J. Neurosci.* 32 12973–12978. 10.1523/jneurosci.1597-12.2012 22973021 PMC3478885

[B62] Reynolds I. J. Hastings T. G. (1995). Glutamate induces the production of reactive oxygen species in cultured forebrain neurons following NMDA receptor activation. *J. Neurosci.* 15 3318–3327. 10.1523/jneurosci.15-05-03318.1995 7751912 PMC6578215

[B63] Sacktor T. C. Osten P. Valsamis H. Jiang X. Naik M. U. Sublette E. (1993). Persistent activation of the zeta isoform of protein kinase C in the maintenance of long-term potentiation. *Proc. Natl. Acad. Sci. U.S.A.* 90 8342–8346. 10.1073/pnas.90.18.8342 8378304 PMC47352

[B64] Sanz-Clemente A. Matta J. A. Isaac J. T. Roche K. W. (2010). Casein kinase 2 regulates the NR2 subunit composition of synaptic NMDA receptors. *Neuron* 67 984–996. 10.1016/j.neuron.2010.08.011 20869595 PMC2947143

[B65] Sattler R. Charlton M. P. Hafner M. Tymianski M. (1998). Distinct influx pathways, not calcium load, determine neuronal vulnerability to calcium neurotoxicity. *J. Neurochem.* 71 2349–2364. 10.1046/j.1471-4159.1998.71062349.x 9832133

[B66] Sattler R. Xiong Z. Lu W. Y. Hafner M. Macdonald J. F. Tymianski M. (1999). Specific coupling of NMDA receptor activation to nitric oxide neurotoxicity by PSD-95 protein. *Science* 284 1845–1848. 10.1126/science.284.5421.1845 10364559

[B67] Sengpiel B. Preis E. Krieglstein J. Prehn J. H. (1998). NMDA-induced superoxide production and neurotoxicity in cultured rat hippocampal neurons: role of mitochondria. *Eur. J. Neurosci.* 10 1903–1910. 10.1046/j.1460-9568.1998.00202.x 9751160

[B68] Snezhkina A. V. Kudryavtseva A. V. Kardymon O. L. Savvateeva M. V. Melnikova N. V. Krasnov G. S. (2019). ROS generation and antioxidant defense systems in normal and malignant cells. *Oxid. Med. Cell Longev.* 2019:1–17. 10.1155/2019/6175804 31467634 PMC6701375

[B69] Stanika R. I. Pivovarova N. B. Brantner C. A. Watts C. A. Winters C. A. Andrews S. B. (2009). Coupling diverse routes of calcium entry to mitochondrial dysfunction and glutamate excitotoxicity. *Proc. Natl. Acad. Sci. U.S.A.* 106 9854–9859. 10.1073/pnas.0903546106 19482936 PMC2701040

[B70] Stanika R. I. Villanueva I. Kazanina G. Andrews S. B. Pivovarova N. B. (2012). Comparative impact of voltage-gated calcium channels and NMDA receptors on mitochondria-mediated neuronal injury. *J. Neurosci.* 32 6642–6650. 10.1523/jneurosci.6008-11.2012 22573686 PMC3370824

[B71] Starkov A. A. Chinopoulos C. Fiskum G. (2004). Mitochondrial calcium and oxidative stress as mediators of ischemic brain injury. *Cell Calcium* 36 257–264. 10.1016/j.ceca.2004.02.012 15261481

[B72] Stein I. S. Park D. K. Flores J. C. Jahncke J. N. Zito K. (2020). Molecular mechanisms of non-ionotropic NMDA receptor signaling in dendritic spine shrinkage. *J. Neurosci.* 40 3741–3750. 10.1523/jneurosci.0046-20.2020 32321746 PMC7204083

[B73] Tamburri A. Dudilot A. Licea S. Bourgeois C. Boehm J. (2013). NMDA-receptor activation but not ion flux is required for amyloid-beta induced synaptic depression. *PLoS One* 8:e65350. 10.1371/journal.pone.0065350 23750255 PMC3672194

[B74] Tejada-Simon M. V. Serrano F. Villasana L. E. Kanterewicz B. I. Wu G. Y. Quinn M. T. (2005). Synaptic localization of a functional NADPH oxidase in the mouse hippocampus. *Mol. Cell Neurosci.* 29 97–106. 10.1016/j.mcn.2005.01.007 15866050 PMC2013304

[B75] Traynelis S. F. Wollmuth L. P. Mcbain C. J. Menniti F. S. Vance K. M. Ogden K. K. (2010). Glutamate receptor ion channels: structure, regulation, and function. *Pharmacol. Rev.* 62 405–496. 20716669 10.1124/pr.109.002451 PMC2964903

[B76] Tymianski M. Charlton M. P. Carlen P. L. Tator C. H. (1993). Source specificity of early calcium neurotoxicity in cultured embryonic spinal neurons. *J. Neurosci.* 13 2085–2104. 10.1523/jneurosci.13-05-02085.1993 8097530 PMC6576557

[B77] Vissel B. Krupp J. J. Heinemann S. F. Westbrook G. L. (2001). A use-dependent tyrosine dephosphorylation of NMDA receptors is independent of ion flux. *Nat. Neurosci.* 4 587–596. 10.1038/88404 11369939

[B78] Wang G. Coleman C. G. Chan J. Faraco G. Marques-Lopes J. Milner T. A. (2013a). Angiotensin II slow-pressor hypertension enhances NMDA currents and NOX2-dependent superoxide production in hypothalamic paraventricular neurons. *Am. J. Physiol. Regul. Integr. Comp. Physiol.* 304 R1096–R1106. 23576605 10.1152/ajpregu.00367.2012 PMC3680791

[B79] Wang J. Q. Chen Q. Wang X. Wang Q. C. Wang Y. Cheng H. P. (2013b). Dysregulation of mitochondrial calcium signaling and superoxide flashes cause mitochondrial genomic DNA damage in Huntington disease. *J. Biol. Chem.* 288 3070–3084. 10.1074/jbc.m112.407726 23250749 PMC3561531

[B80] Wang Y. B. Wang J. J. Wang S. H. Liu S. S. Cao J. Y. Li X. M. (2012). Adaptor protein APPL1 couples synaptic NMDA receptor with neuronal prosurvival phosphatidylinositol 3-kinase/Akt pathway. *J. Neurosci.* 32 11919–11929. 10.1523/jneurosci.3852-11.2012 22933778 PMC6621526

[B81] Watanabe M. Inoue Y. Sakimura K. Mishina M. (1993). Distinct distributions of five N-methyl-D-aspartate receptor channel subunit mRNAs in the forebrain. *J. Comp. Neurol.* 338 377–390. 10.1002/cne.903380305 8113446

[B82] Weilinger N. L. Lohman A. W. Rakai B. D. Ma E. M. Bialecki J. Maslieieva V. (2016). Metabotropic NMDA receptor signaling couples Src family kinases to pannexin-1 during excitotoxicity. *Nat. Neurosci.* 19 432–442. 10.1038/nn.4236 26854804

[B83] Weilinger N. L. Tang P. L. Thompson R. J. (2012). Anoxia-induced NMDA receptor activation opens pannexin channels via Src family kinases. *J. Neurosci.* 32 12579–12588. 10.1523/jneurosci.1267-12.2012 22956847 PMC6621249

[B84] Yeung A. K. Patil C. S. Jackson M. F. (2020). Pannexin-1 in the CNS: emerging concepts in health and disease. *J. Neurochem.* 10.1111/jnc.15004 online ahead of print, 32162337

[B85] Zorov D. B. Juhaszova M. Sollott S. J. (2006). Mitochondrial ROS-induced ROS release: an update and review. *Biochim. Biophys. Acta* 1757 509–517. 10.1016/j.bbabio.2006.04.029 16829228

